# Endogenous Lipid Signals in Energy Homeostasis and Fibrosis

**DOI:** 10.3390/biom16081170

**Published:** 2026-08-11

**Authors:** Camilla Di Meo, Sakthimala Palaniappan, Cristina Urbano, Giacomo Cimino, Francesco Cestra, Noemi De Dominicis, Veronica Carnicelli, Annamaria Tisi, Mauro Maccarrone

**Affiliations:** 1 Department of Biotechnological and Applied Clinical Sciences, University of L’Aquila, 67100 L’Aquila, Italy; camilla.dimeo@univaq.it (C.D.M.); sakthimala.palaniappan@graduate.univaq.it (S.P.); cristina.urbano@graduate.univaq.it (C.U.); giacomo.cimino@graduate.univaq.it (G.C.); cestrafrancesco02@gmail.com (F.C.); veronica.carnicelli@univaq.it (V.C.); 2 LIFE Institute for Research and Health Care, Santa Lucia Istituto di Ricovero e Cura a Carattere Scientifico (IRCCS), 00143 Rome, Italy; noemi.dedominicis@student.univaq.it

**Keywords:** bioactive lipids, fatty acid mediators, eicosanoids, specialized pro-resolving mediators, endocannabinoid system, energy homeostasis, fibrosis

## Abstract

Endogenous bioactive lipids are complex signaling mediators actively involved in a plethora of pathophysiological processes, including energy homeostasis and fibrosis. Energy homeostasis is a stable internal state resulting from a dynamic balance between energy expenditure and storage. Dysregulated energy balance can contribute to fibrosis, exhibiting key metabolic effects on insulin sensitivity, glucose tolerance, lipid accumulation and metabolism, as well as on energy expenditure, ultimately leading to metabolic disorders. In this context, three major classes of endogenous lipid mediators derived from polyunsaturated fatty acids (PUFAs)—eicosanoids, specialized pro-resolving mediators, and endocannabinoids—represent a key signaling network involved in the regulation of energy metabolism. Hence, alterations in their metabolism and signaling are often associated with fibrosis and other related molecular changes. Here, we provide a comprehensive overview of the role of the above-mentioned lipid classes in energy homeostasis and fibrosis by reviewing the available literature spanning nearly four decades, with a primary focus on studies published over the past 20 years.

## 1. Introduction

**Figure 1:**
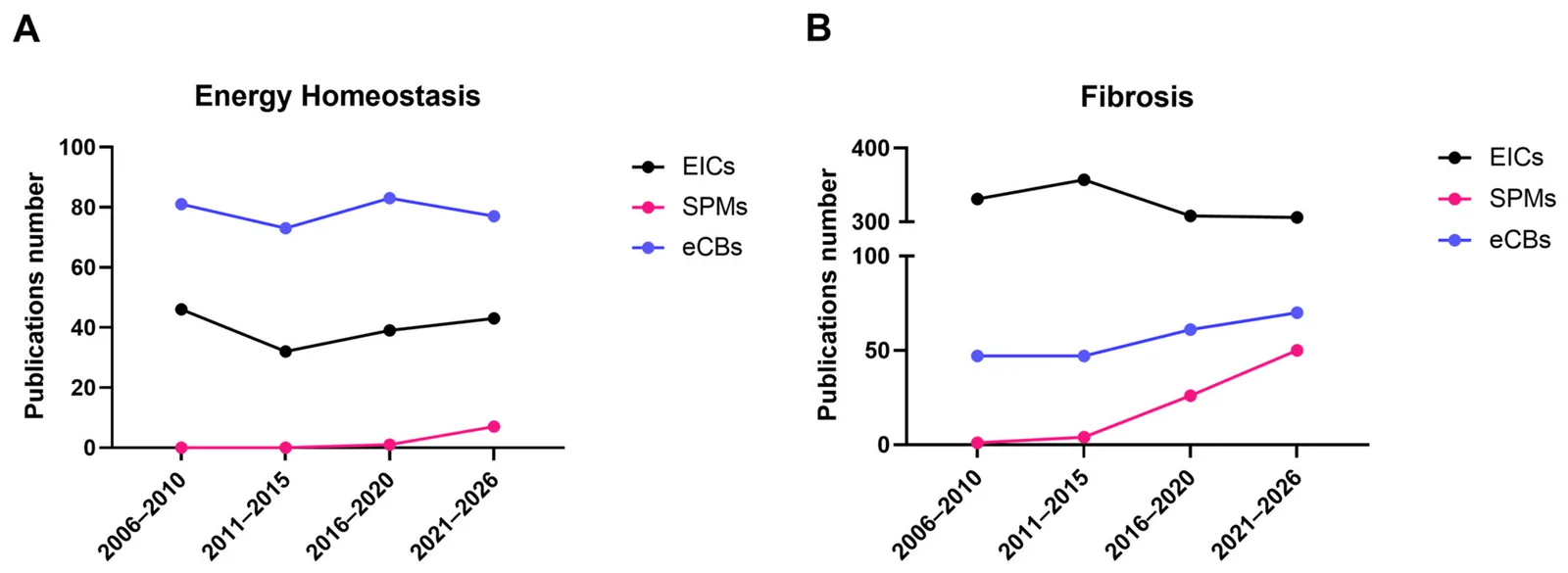
Number of publications on bioactive lipids in energy homeostasis and fibrosis over the last 20 years. The number of PubMed-indexed publications in 5-year intervals, from 2006 to 2026, is shown for eicosanoids (EICs; black line), specialized pro-resolving mediators (SPMs; pink line), and endocannabinoids (eCBs; blue line), in relation to energy homeostasis (**A**) and fibrosis (**B**).

Energy homeostasis is a fundamental physiological process responsible for the maintenance of energy balance in our body and is tightly regulated by the coordinated interplay of multiple organs, including adipose tissue, brain, and gastrointestinal tract [1]. Any alterations in these processes may lead to pathological events, including fibrosis, where aberrant and uncontrolled remodeling of the extracellular matrix (ECM) occurs [2]. In this context, a critical role is played by endogenous lipid signals, particularly those derived from polyunsaturated fatty acids (PUFAs). Among these, eicosanoids (EICs), specialized pro-resolving lipid mediators (SPMs), and endocannabinoids (eCBs) are particularly relevant in integrating inflammatory and metabolic signals to preserve tissue homeostasis, and their dysregulation has been associated with a number of diseases [3,4,5]. Despite the large number of studies and the growing interest in this field, a comprehensive and integrated review of the roles of these bioactive lipids in energy homeostasis and fibrosis is still lacking. Building on this, in the present review, we aimed to provide a comprehensive and up-to-date state-of the art on the metabolism and signaling of EICs, SPMs, and eCBs, key players in energy homeostasis and fibrosis. To this end, we reviewed articles published over the last four decades using PubMed as the literature database. The interest in these topics is reflected by the steadily large number of studies published in the field—particularly on fibrosis—during the last 20 years (Figure 1). Regarding energy homeostasis (Figure 1A), the number of studies on EICs and eCBs has remained rather similar over time, although research on eCBs has consistently been more intense. Instead, only a limited number of studies have investigated SPMs in the context of energy homeostasis, and these have mainly appeared within the past 5–6 years. As for fibrosis (Figure 1B), the largest research interest has focused by far on EICs, with studies on eCBs and even more on SPMs showing a clear increase over time.

In the following sections, we discuss the general concepts of energy homeostasis and fibrosis, the biochemistry of bioactive lipids, and their involvement in these pathophysiological processes.

## 2. Energy Homeostasis and Fibrosis

Energy homeostasis is a complex process responsible for maintaining energy balance in the body by controlling energy intake, metabolism, storage, and expenditure [1]. Accordingly, energy homeostasis is systemically regulated by several organs—including the brain, adipose tissue, and gastrointestinal tract—through multiple interconnected molecular pathways that involve primarily hormones, but also other mediators such as fatty acids and microbiota-derived metabolites [6,7]. The gastrointestinal system is responsible for nutrient sensing and absorption, hence playing a key role in metabolic balance [7]. Energy storage is mediated by the white adipose tissue (WAT), mainly via the accumulation of triglyceride (TG)-enriched lipid droplets. When energy supply exceeds demand, WAT mass increases; conversely, when energy availability is insufficient, fatty acids may be released by the WAT, providing the required energy. In this context, the adipocyte-derived hormones leptin and adiponectin are pivotal in orchestrating the WAT-associated regulation of energy homeostasis [1]. All these events are finely regulated by the brain, which controls appetite and body weight through a complex neurocircuitry that engages the hypothalamus [8], the brainstem [9], the mesolimbic dopamine circuit [10], and the cognitive frontal and cortical system [11]. Importantly, the gut–brain axis has recently emerged as a key regulatory component of energy homeostasis, whereby signaling molecules may be released by the gastrointestinal tract and reach the brain through the circulation [7].

Dysregulation of energy metabolism contributes to fibrosis, as observed in obesity, where the adipose tissue becomes inflamed and fibrotic [12]. Fibrosis is a process characterized by uncontrolled wound-healing of an injured tissue, which ultimately leads to altered remodeling of the ECM with scarring and wound formation in all the tissues of the human body [2]. For instance, in obesity, WAT undergoes structural and functional remodeling, characterized by massive expansion and secretome alterations, with a subsequent detrimental impact on energy homeostasis [13]. Likewise, energy metabolism is negatively affected when fibrosis develops in organs like the kidney, liver, heart, and others, in the course of different diseases [14,15,16]. For instance, fatty acid oxidation is reduced in kidney fibrosis, leading to aberrant energy metabolism [14]. Similarly, liver fibrosis, which often progresses to cirrhosis and/or cancer, inevitably impacts body energy homeostasis. Yet, the autophagy pathway seems to be fundamental in this process, since its dysregulation leads to hepatic fibrosis and aberrant energy metabolism [16].

Overall, the available literature indicates a close relationship between energy homeostasis and fibrosis. Consistently, energy imbalance may induce organ fibrosis, which in turn further contributes to altered energy homeostasis, thus leading to a detrimental vicious cycle.

## 3. Endogenous Bioactive Lipids

Bioactive lipids constitute a family of endogenous mediators, derived from polyunsaturated fatty acids (PUFAs). To date, several sub-families of bioactive lipids have been identified, and here we focus on the three major classes characterized so far: EICs, SPMs, and eCBs. The metabolism and signaling of these mediators have been shown to be involved in multiple pathophysiological processes, including energy homeostasis and fibrosis. In the following sections, we review the metabolism and signaling of each class, along with their impact on energy homeostasis (summarized in Table 1) and fibrosis (summarized in Table 2).

## 4. Eicosanoids (EICs)

The EICs are a well-characterized class of fatty acid mediators comprising more than 100 molecules. Depending on their biosynthetic origin and receptor engagement, EICs play a key role in either pro-homeostatic or pathological processes associated with inflammation and fibrosis [110].

### 4.1. Chemistry and Classification of EICs

The EICs are a heterogeneous class of lipids derived from arachidonic acid (AA), a C20:4 *n*–6 (ω–6) PUFA. From a chemical perspective, EICs are oxygenated PUFA derivatives characterized by substantial structural diversity, reflecting differences in ring formation, degree of unsaturation, and oxygenation pattern, which collectively contribute to their distinct biological activities. Depending on the enzymatic route of oxygenation, EICs are classified in the following main families: (i) prostanoids, comprising prostaglandins (PGs) and prostacyclins (PGIs), defined by a cyclopentane ring, and thromboxanes (TXs), containing a six-membered oxane ring; (ii) leukotrienes (LTs) and lipoxins (LXs), which retain the linear AA backbone; (iii) epoxy- (EETs) and hydroxy-eicosatetraenoic acids (HETEs), which preserve the acyclic AA scaffold modified respectively by a single epoxide group or by a hydroxyl group (at the terminal ω-carbon, for 20-HETE) [111]. In addition, it is worth mentioning that other EICs (e.g., PGI_3_, TXA_3_, PGE_3_, LTB_5_) may also be generated by EPA oxygenation, supporting different biological functions [112].

### 4.2. Metabolism and Signaling of EICs

In response to several external stimuli, AA is released from the phospholipid bilayer through the hydrolysis of the ester bond by phospholipase (PL) A_2_, thus initiating the signaling cascade [113]. The biosynthesis of EICs primarily follows three distinct enzymatic routes: (i) the cyclooxygenase (COX)-mediated pathway [114], (ii) the lipoxygenase (LOX)-mediated pathway [115,116], and (iii) the cytochrome P450 (CYP450) epoxygenase pathway [117,118].

COX-derived EICs comprise prostanoids (PGs, PGIs, TXs). The isoforms COX-1 and COX-2 convert AA into the common intermediate PGH_2_, which is subsequently metabolized by tissue-specific isomerases to generate prostanoids like PGE_2_, PGF_2α_, PGD_2_, PGI_2_, and TXA_2_. Of interest, PGD_2_ undergoes non-enzymatic degradation in plasma, generating metabolites like PGJ_2_ that retain biological activity [119].

LOX-derived eicosanoids include LTs (LTA_4_, LTB_4_) and cysteinyl LTs (i.e., LTC_4_, LTD_4_, LTE_4_), along with 12-oxoETEs (HxA and HxB) and LXs (LXA_4_ and LXB_4_). The LOX pathway involves 5-LOX, 12-LOX, and 15-LOX, which convert AA into 5-hydroperoxyeicosatetraenoic acid (HpETE)—precursors of LTs-, 12-HpETE—precursor of 12-oxoETEs and hepoxilins—and 15-HpETE—precursor of LXs-, respectively.

Finally, the CYP450 pathway generates hydroxy- and epoxy-derivatives of AA, which include the EETs and 20-HETE [117].

As for the degradative pathways of EICs, their catabolism is primarily mediated by enzymes such as 15-hydroxyprostaglandin dehydrogenase (15-PGDH), soluble epoxide hydrolase (sEH), prostaglandin reductase (PTGR), and 11-hydroxythromboxane B2 reductase (11-TXDH), with pathway-specific mechanisms depending on the EIC class [120]. Additionally, EICs can undergo further biotransformations, including β-oxidation, CYP 450 ω-hydroxylation, and glucuronidation [120].

Collectively, these molecules act through specific G-protein coupled receptors (GPCRs) to regulate a wide range of physiological and pathological processes [114]. Among these, LOX-derived EICs such as LTB_4_ bind to BLT1 and BLT2, and others like CysLTs target CysLTR1 and CysLTR2, [115,116]. Of note, despite sharing AA as a common precursor, individual EIC classes like LTs and LXs exert opposing biological functions [121], depending on their biosynthetic origin and receptor engagement, as further discussed in Section 4.3 and Section 5.2.

**Figure 2:**
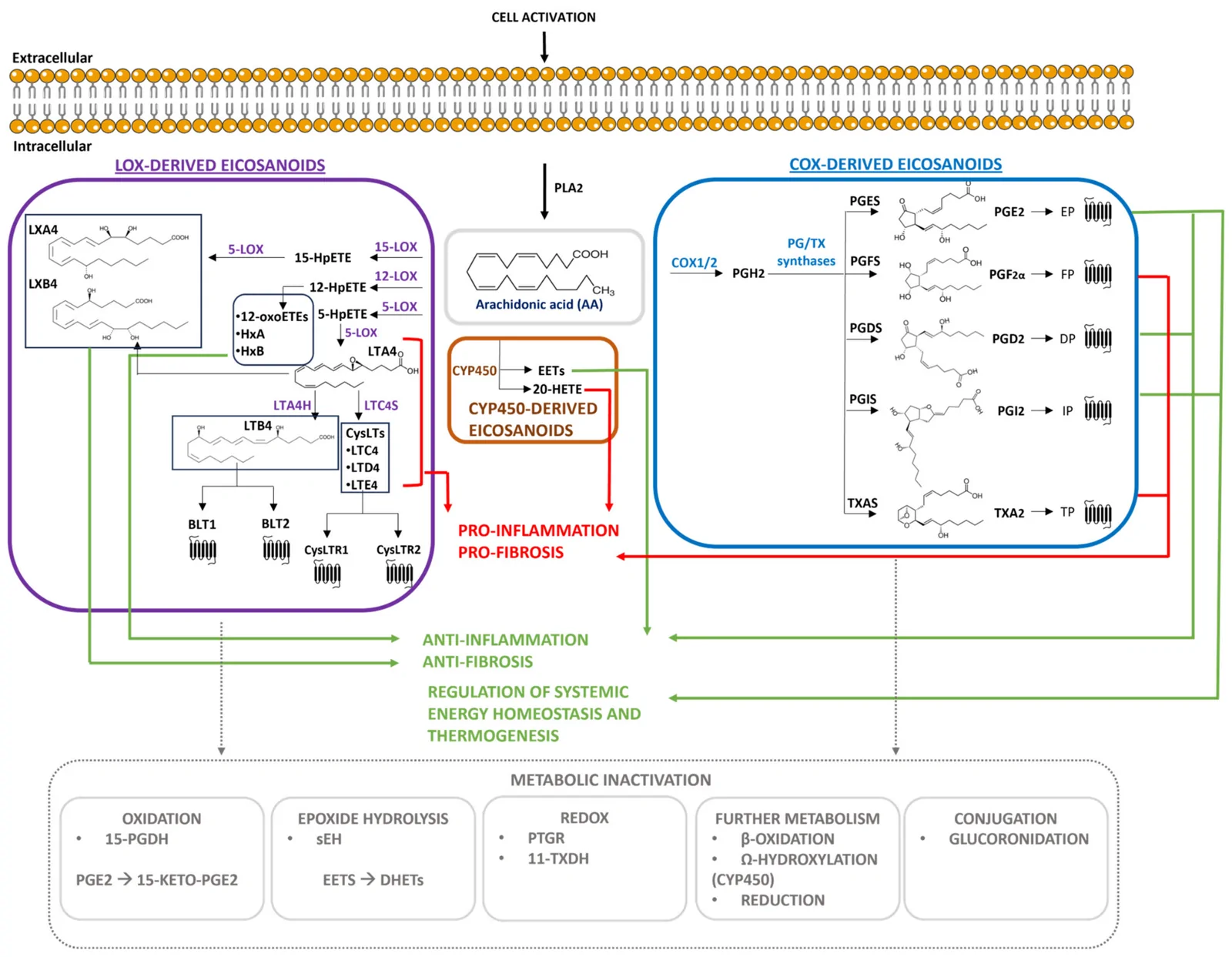
Metabolism, receptors and signaling of eicosanoids (EICs), and their role in energy homeostasis and fibrosis. The figure reports the major biosynthetic pathways and known receptors of the three EIC classes (LOX-derived, COX-derived and CYP450-derived EICs), that are involved in (i) pro-inflammatory and pro-fibrotic processes leading to unfavorable homeostatic conditions (denoted by the red arrows), or (ii) anti-inflammatory and anti-fibrotic processes (denoted by the green arrows) that establish favorable bioenergetics. Dashed grey arrows indicate the major metabolic inactivation routes for EICs.

More details on the metabolism and signaling of EICs beyond those summarized in Figure 2 can be found elsewhere [122].

### 4.3. EICs in Energy Homeostasis and Fibrosis

The EICs are involved in the regulation of energy homeostasis [17,18,19,25,27,29,34,35,36,37,40,41,123], and in controlling pro-fibrotic and anti-fibrotic processes throughout the body [124,125,126,127]. Indeed, they are generated at all stages of tissue repair under stress conditions in a time-dependent manner. Yet, their biological activity is highly complex, as they may exert opposing effects depending on their biosynthetic pathways and receptor interactions [19].

For instance, imbalance of EICs metabolism and signaling is often associated with fibrosis, scars, and wounds [40,48,49]. Nonetheless, some EICs, such as PGE_2_, PGD_2_, PGI_2_, LXs, hepoxilins, and EETs, show anti-fibrotic properties due to the inhibition of the transforming growth factor (TGF)-β pathway, the major driver of fibrosis. The main effects of TGF-β inhibition are associated with anti-inflammatory processes and blocking of fibroblast proliferation and myofibroblast differentiation, which are all important events contributing to tissue fibrosis [3,61,63,66,127,128,129]. Yet, LXs have demonstrated beneficial effects across multiple organs [130], including liver [62,90,131,132,133], kidney [30,134], lung [31,32,135,136], eye [33] and knee joints [137], by promoting anti-inflammatory and anti-fibrotic processes [138]. Therefore, LXs are also classified among the specialized pro-resolving lipid mediators (SPMs). Additionally, 15-deoxy-Δ12,14 (15d)-PGJ_2_—a bioactive dehydrated downstream metabolite of PGD_2_—has been shown to promote adipocyte differentiation by binding to the PPARγ receptor and to be increased in the plasma of diabetic patients. Of note, these downstream metabolites, which are biologically active, may be of particular interest as potential biomarkers of diseases [119].

Against this background, therapeutic strategies that target the EICs have been developed to counteract tissue fibrosis and inflammation in a wide range of diseases. For instance, ONO-1301, a synthetic PGI_2_, showed preclinical efficacy in attenuating cardiac fibrosis [139] and, importantly, exhibited protective effects against ischemic cardiomyopathy in humans (phase I/II clinical trials) [140]. The same compound also prevented inflammation in cultured macrophages, as well as the occurrence and progression of liver cancer in a mouse model of non-alcoholic steatohepatitis (NASH) [141]. Another prostacyclin analogue, treprostinil, attenuated pulmonary fibrosis and inflammation in preclinical models of mice [142] and rats [143], although clinical data remain heterogeneous [144]. Finally, MRE-269 (or ACT-333679), the active metabolite of the PGI_2_-receptor (IP) agonist selexipag, exerted an anti-fibrotic action in idiopathic pulmonary fibrosis (IPF) [145] and displayed beneficial and anti-proliferative effects in an in vitro model of pulmonary hypertension [146].

Some EICs display pro-inflammatory and pro-fibrotic properties. Among these, PGF_2α_ promotes pulmonary fibrosis via a TGF-β-independent mechanism that engages the PGF_2α_/FPr receptor axis [57]. TXA_2_ has shown COX-1-associated pro-fibrotic and pro-inflammatory effects in mice with inflammatory bowel disease (IBD) [60]. 20-HETE exerts a peculiar action in the kidney as it can either promote renal injury and fibrosis or regulate homeostatic processes [67,68]. Also, LTs have shown pro-inflammatory and pro-fibrotic activities by enhancing TGF-β signaling and increasing extracellular matrix (ECM) deposition [64]. Accordingly, LTs were found to be increased in animal models and patients with IPF [64,147]. Moreover, genetic ablation of BLT1 led to impaired lung fibrosis in mice with bleomycin-induced lung fibrosis, which was attenuated by treatment with BLT1 antagonists in the same animal model [65]. However, it is worth mentioning that the LTB_4_/BLT1 axis has been recently associated with NLRP3 inhibition and subsequent pro-homeostatic effects in the context of influenza-induced lung inflammation [39]. Therefore, the context-dependent role of LTB_4_/BLT1 in tissue homeostasis and fibrosis seems apparent.

Interestingly, several pro-inflammatory PGs, LTs, and TXs have also been associated with the inflammatory response and pulmonary fibrosis in severe coronavirus disease 2019 (COVID-19). Conversely, anti-inflammatory EICs, such as EETs, have been suggested as a potential therapeutic approach to promote resolution of inflammation and contrast organ damage in COVID-19 patients [65].

Finally, among EPA-derived EICs, PGI_3_ is an anti-aggregation and vasodilatory mediator, whereas TXA_3_, PGE_3_ and LTB_5_ display attenuated pro-aggregation and inflammatory potency compared with their AA-derived (series-2/4) counterparts [112]. However, their specific contribution to fibrotic remodeling and energy homeostasis remains largely unexplored, in contrast to the extensively documented roles of their AA-derived counterparts PGE_2_ and LTB_4_.

## 5. Specialized Pro-Resolving Lipid Mediators (SPMs)

The SPMs are a large family of bioactive lipids derived from ω–3 PUFAs and, to a smaller extent, from AA [148]. They have been recently identified as “orchestrators” of resolution of inflammation, an active process leading to the shutdown of inflammatory responses [149].

### 5.1. Chemistry and Classification of SPMs

SPMs are an enzymatically oxygenated class of bioactive lipid autacoids, which are commonly classified into four main chemical families, according to their PUFA precursors: (i) EPA-derived (C20:5 ω–3) compounds, which include E-series resolvins (RvE1–3), characterized by a 20-carbon chain with stereospecific 18(R) or 18(S) hydroxyl groups and conjugated polyene sequences [150,151], along with other metabolites such as PGI_3_, TXA_3_, PGE_3_, LTB_5_ [112]; (ii) DHA-derived (C22:6 ω–3) compounds, comprising a large and structurally heterogeneous group based on a 22-carbon backbone, including D-series resolvins (RvD1–6), maresins (MaR1, 2) and their disulfide conjugates (MCTR1-3), as well as (neuro)protectins (PD1/NPD1 and PDX) and their disulfide conjugates (PCTRs) [152]; (iii) *n*–3 DPA-derived (C22:5 ω–3) compounds, that represent a distinct 22-carbon class featuring 13-series resolvins (RvTs) and *n*–3 DPA analogues of DHA-derived SPMs (${\mathrm{RvD}}_{n-3\;\mathrm{DPA}}$, ${\mathrm{PD}}_{n-3\;\mathrm{DPA}}$, and ${\mathrm{MaR}}_{n-3\;\mathrm{DPA}}$) [152,153]; and (iv) AA-derived compounds, including LXs (e.g., LXA_4_ and LXB_4_) [154]. Each class follows a distinct biosynthetic route, detailed in Section 5.2.

### 5.2. Metabolism and Signaling of SPMs

The SPMs share key biosynthetic routes with EICs, originating from the PLA_2_-mediated release of PUFAs from membrane phospholipids. The latter compounds are subsequently converted through the coordinated action of COX-2, LOXs, and CYP450s, according to the selective SPM class.

In particular, EPA is metabolized by COX-2 or CYP450 to produce the epimers 18(*R*/*S*)-hydroperoxyeicosapentaenoic acid (HpEPE), which follow a similar metabolic route, generating RvE1 and RvE2 through the activity of 5-LOX, peroxidase and hydrolase, or RvE3 through the activity of 12-/15-LOX. Notably, EPA is also the precursor of non-SPM oxygenated metabolites generated via the same COX and 5-LOX pathways, including PGI_3_, TXA_3_, PGE_3_ and LTB_5_ [112].

DHA is produced by the action of 12-/15-LOX, generating 14(*S*)-hydroperoxy-docosapentaenoic acid (14(*S*)-HpDHA) and 17(*S*)-hydroperoxy-docosapentaenoic acid (17(*S*)-HpDHA), respectively. 14(*S*)- HpDHA and 17(*S*)- HpDHA lead to the biosynthesis of MaRs and their disulfide conjugates, along with that of (neuro)protectins and their disulfide conjugates and resolvins (RvD1–5), respectively. Of interest, PD1 biosynthesis is constrained by a critical redox branch point: the precursor 17(*S*)-HpDHA can either be converted to the 16S,17S-epoxide intermediate required for PD1 formation, or rapidly reduced by glutathione peroxidase 4 (GPx4) to 17(*S*)-HDHA, which is no longer competent for epoxide formation [155]. Instead, PDX is biosynthesized via a double oxygenation of DHA, less dependent on the epoxide intermediate, and is often detected more readily than PD1 in biological samples [155].

The *n*–3 DPA generates 13*R*-HpDPA*_n_*_–3_ (a precursor of RvT1-4), 14-HpDPA (a precursor of MaR1–3*_n_*_–3 DPA_) and 17-HpDPA (a precursor of PD1-2*_n_*_–3 DPA_, RvD1–2*_n_*_–3 DPA_ and RvD5*_n_*_–3 DPA_) through the action of COX-2, 12-LOX and 15-LOX, respectively [156,157,158].

Lastly, LXs [154] biosynthesis involves the sequential action of 15-LOX, which converts AA into 15-HpETE, followed by further processing mediated by 5-LOX.

These mediators signal through a diversified GPCR network. In particular, EPA-derived SPMs exert their biological effects primarily through the ChemR23 receptor [159], although RvE1 and RvE2 can also antagonize the pro-inflammatory BLT1 receptor [160]. The landscape of GPCRs engaged by DHA derivatives includes: FPR2/ALX, targeted by RvD1, AT-RvD1 and RvD3; GPR18, targeted by RvD2; GPR32, targeted by RvD1, AT-RvD1, RvD3 and RvD5; GPR37, targeted by PD; and LGR6 targeted by Mar1 [152]. Finally, both LXA_4_ and LXB_4_ exert their biological effects by binding to FPR2/ALX, which is also a target of RvDs, as reported above.

To date, the only known catabolic enzyme of SPMs is 15-PGDH, yet its specific role in SPM inactivation remains to be fully elucidated [161].

**Figure 3:**
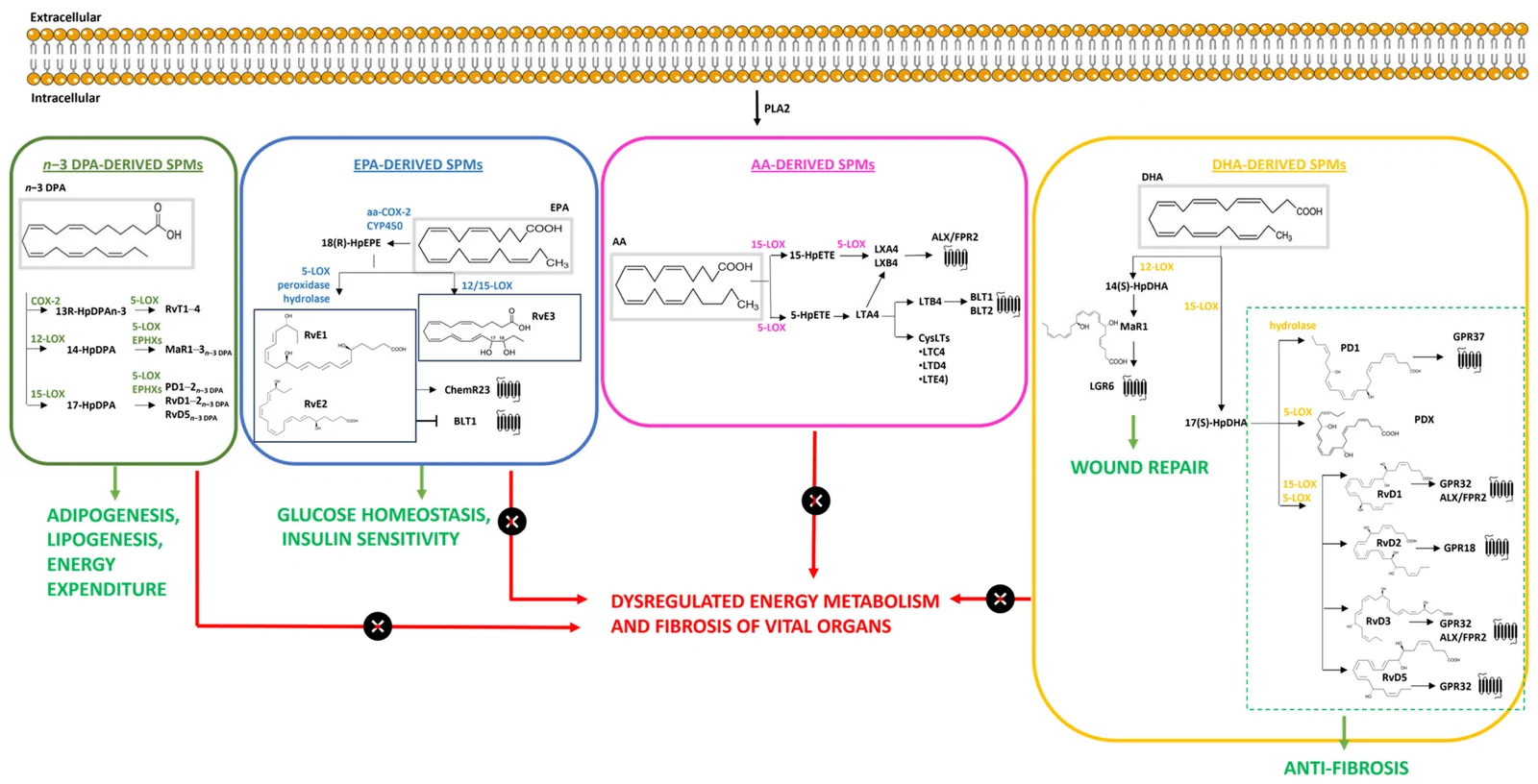
Metabolic pathways and known receptors of specialized pro-resolving lipid mediators (SPMs) and their role in energy homeostasis and fibrosis. The figure reports the major biosynthetic pathways and known receptors of the main SPM classes: *n*–3 DPA-derived, EPA-derived, AA-derived, and DHA-derived SPMs. SPM metabolism and signaling are involved in maintaining energy homeostasis (indicated by the green arrows), and any alterations/impairments of physiological signalling mechanisms can lead to dysregulated energy metabolism and fibrotic processes (indicated by the red arrows crossed by an “×”).

An overview of the biosynthetic pathways and known receptors of the major SPMs is shown in Figure 3.

### 5.3. SPMs in Energy Homeostasis and Fibrosis

Inflammation is an important regulator of energy homeostasis [162] and fibrogenesis [163]. Consequently, SPMs play a major role in these processes and related metabolic diseases [164], being key orchestrators of resolution of inflammation. In this context, accumulated evidence from in vitro and in vivo studies indicates that *n*–3 PUFA-derived SPMs are critically involved in the regulation of energy homeostasis, particularly by promoting homeostatic adipocyte regulation and energy expenditure [48]. Among these mediators, the RvE1-ChemR23 axis has been shown to regulate glucose homeostasis and insulin sensitivity by controlling the transcriptional profile in the liver of obese mice [43,165]. In line with these experimental findings, a large human cohort study reported that circulating RvE1 levels are inversely associated with multiple indices of adiposity, including body mass index (BMI), waist circumference, and subcutaneous fat volume, and are significantly reduced in obese compared with normal-weight subjects, supporting a potential link between RvE1 deficiency and obesity-associated chronic low-grade inflammation [166]. In addition, RvE1 was also shown to mitigate the progression of several fibrosis-related diseases, including interstitial fibrosis in a mouse model [70] and liver fibrosis in both rat [167] and mouse [71] models.

Several studies have also demonstrated beneficial effects of exogenous administration of SPMs across different pathological conditions [168,169,170,171]. For instance, RvD1 attenuated energy metabolism dysregulation associated with lung ischemia/reperfusion injury in rats [170,172] and showed positive outcomes in multiple liver diseases [173], such as non-alcoholic fatty liver disease (NAFLD) [169], liver ischemia/reperfusion injury [168], and acute liver injury [172,173]. However, although a few studies have specifically addressed its role in liver fibrosis, available evidence suggests context-dependent effects [90,174]. Notably, recent findings indicate that RvD1 exerts a protective effect against hepatic fibrosis, preventing inflammation in both mouse and human hepatic stellate cell models, partly via SIRT1-mediated NF-κB and TGF-β1/Smads pathways [175].

Interestingly, emerging evidence also supports the involvement of SPMs in cystic fibrosis (CF), a condition characterized by chronic, non-resolving inflammation. Although it remains unclear whether CF pathogenesis is associated with increased or decreased SPM levels [176,177] RvD1 was found to improve inflammation in primary human cells and transgenic mouse models of CF, holding promise for lung inflammatory diseases [178].

Furthermore, RvE3 has been demonstrated to increase insulin sensitivity, with an upregulation of glucose uptake by activating the PI3K/Akt signaling pathways in adipocytes of mice with diet-induced obesity [179]. Similarly, MaR1 drives tissue regeneration, suggesting that any alterations of its metabolism/signaling may contribute to fibrosis, scars, and impaired wound healing [180]. In addition, MaR1 has been shown to regulate energy homeostasis, since its administration in vivo ameliorates insulin sensitivity and exerts beneficial effects in obesity-associated inflammatory processes in murine models [45,181].

Overall, unresolved inflammation and deregulated metabolism and signaling of SPMs contribute to tissue damage repair, imbalance of energy metabolism, and fibrosis in multiple organs, contributing to liver pathologies [72,74,182], kidney disease [82], as well as renal [73,182] and cardiac fibrosis [75,182,183].

## 6. Endocannabinoids (eCBs)

The eCBs are a class of ubiquitous lipid signals, which bind to cannabinoid receptors 1 and 2 (CB_1/2_) and trigger biological effects similar to those triggered by Δ^9^-tetrahydrocannabinol (THC), the main psychoactive component of *Cannabis sativa* and *Cannabis indica* plants [184]. These mediators play manifold roles, both centrally and peripherally, in different physiological and pathological processes [185].

### 6.1. Chemistry and Classification of eCBs

The so-called “endocannabinoid system” (ECS) comprises eCBs, their biosynthetic and degradative enzymes, receptor targets and membrane transporters. Chemically, eCBs are derived from long-chain PUFAs—in particular arachidonic acid (AA, C20:4 *n*–6)—linked to ethanolamine or glycerol headgroups. The major eCBs belong to two distinct chemical classes: (i) *N*-acylethanolamines (NAEs), represented by *N*-arachidonoylethanolamine or anandamide (AEA), with a fatty acyl chain linked to ethanolamine via an amide bond; and (ii) monoacylglycerols (MAGs), represented by 2-arachidonoylglycerol (2-AG), an ester formed between the hydroxyl group at the *sn*-2 position of glycerol and AA [186,187]. Beyond AEA and 2-AG, the chemical ECS landscape extends to congeners of NAEs known as “eCB-like” compounds, due to their similar chemical structures and partially overlapping signaling pathways; however, detailed information on their metabolism and function is limited compared to classical eCBs [188]. These eCB-like lipids include *N*-palmitoylethanolamine (PEA), *N*-oleoylethanolamine (OEA), *N*-linoleoylethanolamine (LEA), *N*-stearoylethanolamine (SEA), *N*-palmitoleoylethanolamine (POEA), *N*-epoxyeicosatetraenoylethanolamine (EPEA), and *N*-docosahexaenoylethanolamine or synaptamide (DHEA) [188]. Furthermore, enzymatic oxygenation of the arachidonate backbone in AEA and 2-AG generates oxygenated derivatives—including PG-ethanolamides, PG-glyceryl esters, hydroxy-AEAs, and HETE-glycerols—altogether forming an “oxyendocannabinoidome” that has been extensively reviewed elsewhere [83].

### 6.2. Metabolism and Signaling of eCBs

According to their chemical class, eCBs follow selective biosynthetic pathways: AEA is mainly synthesized by *N*-acylphosphatidylethanolamines-specific phospholipase D (NAPE-PLD) [189], whereas 2-AG is primarily produced by *sn*-1-diacylglycerol lipases α and β (DAGL-α/β) [190]. In addition to these main biosynthetic routes, AEA and 2-AG can also be synthesized by phospholipase C (PLC) or αβ-hydrolase domain 4 protein (ABHD4), respectively [185,191]. The primary degradative enzyme for AEA is the fatty acid amide hydrolase (FAAH), which releases AA and ethanolamine (EtNH_2_) [192], whereas 2-AG degradation mainly depends on monoacylglycerol lipase (MAGL), which releases AA and glycerol [165]. Incidentally, FAAH can also break the ester bond of 2-AG, though to a minor extent [165]. Other enzymes involved in eCB catabolism are *N*-acylethanolamine acid amidase (NAAA)—for AEA—and ABHD6/12—for 2-AG [193,194], in addition to other less characterized degradative routes [195]. In addition, AEA and 2-AG can also be oxygenated by COX-2, 12-LOX, 15-LOX, and CYP450, generating the oxidized derivatives—that altogether form the “oxyendocannabinoidome” [83]. Interestingly, the biological functions of many of these oxidized compounds remain largely unknown, and it remains to be clarified whether they act through eCB-target receptors or alternative molecular mechanisms.

The principal eCBs signal primarily through G_i/o_ protein-coupled CB_1_ and CB_2_ [196,197]. In addition, eCBs can bind to and activate the transient receptor potential vanilloid 1 (TRPV1) channels, peroxisome proliferator activated receptors α, γ, δ (PPARα/γ/δ), or the orphan G protein-coupled receptors GPR55 and GPR119 [198].

**Figure 4:**
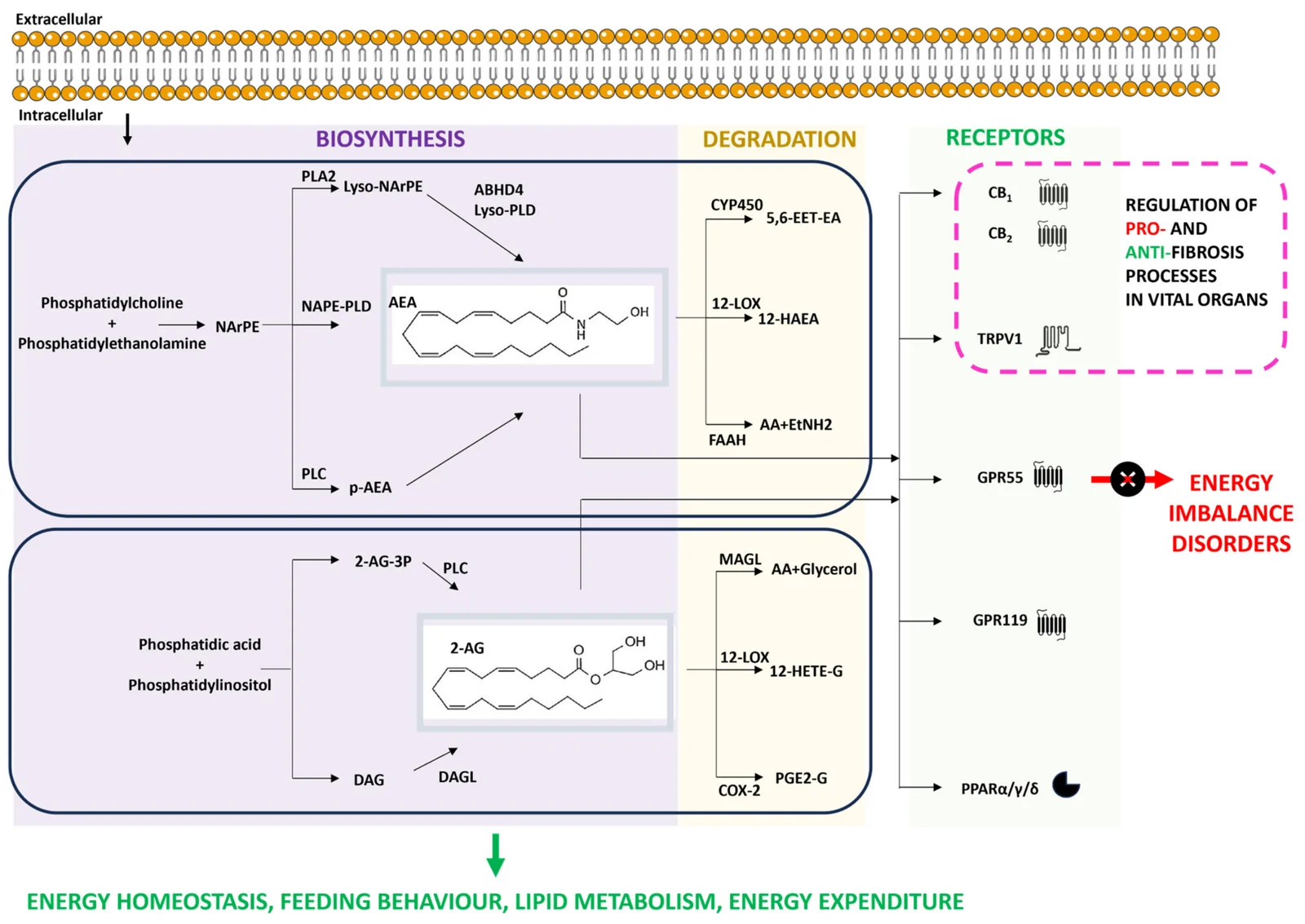
Metabolic pathways and known receptors of endocannabinoids (eCBs) and their role in energy homeostasis and fibrosis. The figure reports the major biosynthetic and degradative pathways, along with known receptors, of the two main eCBs: AEA and 2-AG. eCBs metabolism is essential to maintain homeostatic energy metabolism, storage, and expenditure (indicated by green arrows), and impairment of signaling cascades can lead to energy imbalance disorders (indicated by red arrows crossed by an “×”).

Metabolic pathways and known receptors of eCBs are schematically depicted in Figure 4.

### 6.3. eCBs in Energy Homeostasis and Fibrosis

The eCBs are pivotal regulators of energy homeostasis [199,200], driving feeding behavior [201], lipid metabolism and energy expenditure [4]. Accordingly, the ECS is deeply implicated in diseases characterized by energy imbalance, such as obesity and obesity-related disorders; for comprehensive reviews see refs [49,53,54,55,56]. Within this framework, the CB_1_ receptor has emerged as a primary mediator of metabolic and fibrotic dysfunctions. Indeed, CB_1_-deficient mice exhibited a significant reduction in body weight [202]; similar effects have been observed in humans upon administration of the CB_1_ antagonist/inverse agonist rimonabant [203], yet its clinical use had to be discontinued in 2008 due to severe psychiatric and neurological adverse effects [204]. Moreover, CB_1_ signaling has been consistently associated with fibrogenesis across multiple organs. Interestingly, both genetic deletion and pharmacological inhibition of CB_1_ have been shown to attenuate fibrosis by suppressing key pro-fibrotic markers, including TGF-β and α-smooth muscle actin (α-SMA), in obesity-related liver diseases (e.g., NAFLD) and in hepatic fibrosis mouse models [84,85], indicating that CB_1_ activation promotes fibrotic processes. Consistently, CB_1_ expression increased in human cirrhosis and in activated hepatic stellate cells (HSCs) [84]. Similar effects have been observed in pulmonary fibrosis—including IPF and radiation-induced fibrosis (RIF) [86,87,88]—and cardiac fibrosis [89], and CB_1_ antagonism with MRI-1867 has been shown to reduce lung fibrosis in preclinical models [88].

Conversely, CB_2_ activation exhibits beneficial outcomes in the context of fibrosis and energy homeostasis [205]. For instance, CB_2_ agonism has been shown to induce anti-fibrotic effects in the liver [90,91,92], protection from kidney fibrosis in a model of unilateral ureteral obstruction [93], as well as from pulmonary [94,95] and skin fibrosis [96]. Accordingly, increased liver fibrosis is observed in mice lacking CB_2_ expression [97]. In keeping with these findings, other studies performed on animal models suggest that reduced CB_2_ signaling leads to increased food intake, greater adipocyte mass and obesity, effects that are reverted by CB_2_ agonism [206,207].

Beyond the canonical CB_1/2_ receptors, other eCB targets have been implicated in tissue remodelling. Among these, the TRPV1 channel displays a context-dependent role in tissue repair and fibrosis. In fact, TRPV1 expression is altered in pulmonary fibrosis, increasing in guinea pig models [98] and decreasing in IPF patients [99]. Interestingly, TRPV1 activation appears to exert protective effects in hepatic [100], renal [101,102], and cardiac fibrosis [103,104,105], whereas its inhibition has shown anti-fibrotic effects in a corneal injury mouse model [106], as well as in both in vitro and in vivo IPF models [107]. These observations highlight the dual and context-dependent role of TRPV1.

Nuclear PPARs also play a key role as mediators of energy homeostasis, and thus they have been extensively studied in the context of metabolic dysfunction (extensively reviewed in [108]). For instance, pharmacological activation of PPARs has been shown to (i) regulate lipid metabolism [208], (ii) reduce lipid accumulation in the liver [209], and (iii) exert anti-diabetic effects [209]. Nonetheless, their clinical exploitation remains limited by the occurrence of adverse effects [210].

Finally, it should be mentioned that emerging therapeutic strategies are focusing on minor phytocannabinoids, which exert their biological effects through eCBs-binding receptors like CB_1_, CB_2_, TRPV1 and PPARs. In this context, cannabigerol (CBG) was demonstrated to mitigate hepatic lipid accumulation and inflammation through the AMP-activated protein kinase (AMPK) pathway in human hepatocytes, representing a suitable ECS-oriented therapeutic strategy [109].

## 7. Translational Challenges and Future Directions

From a translational perspective, targeting bioactive lipid signaling has shown promise in experimental models of metabolic and fibrotic diseases, supporting their potential as multi-level therapeutic nodes. In fact, there are available therapeutic approaches targeting EICs, the oldest and best-characterized class of bioactive lipids. For example, non-steroidal anti-inflammatory drugs (NSAIDs) are widely used COX inhibitors that suppress the synthesis of prostanoids and are administered in multiple pathological contexts [211]. Other notable, more recent examples include agonists of EICs (e.g., ONO-1301, MRE-269/ACT-333679) that have shown beneficial effects against cardiac and lung fibrosis, yet their use is still limited to preclinical studies [139,145]. As for the eCBs, although their metabolism and signaling pathways have been largely investigated, to date only a few drugs have been approved for clinical use, and in some cases cytotoxicity and side effects have been observed [212,213]. Moreover, in recent years, phytocannabinoids are gaining particular interest also due to methodological advancements in their extraction and synthesis through microbial engineering (e.g., in *E. coli*, algae, and yeast) [214]. However, even in this case, despite the recognized beneficial effects in multiple pathological models, most of the available evidence derives only from preclinical studies. Finally, owing to the relatively recent discovery of SPMs, evidence supporting their therapeutic application in energy homeostasis and fibrosis remains limited. Likewise, the clinical development of SPM-based therapies or compounds targeting their receptors or biosynthetic enzymes is still in its infancy, with most approaches remaining mostly at the preclinical stages.

Another important aspect to be considered is the limited stability of bioactive lipids. Indeed, these compounds are synthesized on demand and exert their biological activity locally. Likewise, EICs, SPMs and eCBs show very short half-lives, making even their detection in biological samples highly challenging [119,215,216]. For example, TxA_2_ and PGI_2_ exhibit half-lives of 30 s and 2 min, respectively, in circulation [119]. Instead, prostanoid metabolites are relatively more stable and may be more easily detected in urine, thereby providing a more precise and informative source on prostanoid metabolism [119]. Given the highly dynamic nature of bioactive lipid turnover, novel experimental strategies are being developed to monitor real-time changes in lipid signaling, such as fluorescent sensors [217] and mass spectrometry imaging [218] of eCBs. Although these tools are currently limited to experimental models, they represent promising strategies to expand our understanding of lipid metabolism and signaling dynamics with unprecedented temporal resolution.

In the future, further studies aimed at elucidating the selective contribution of these endogenous signals to bioenergetics, as well as their mechanisms of action and integration with other transduction cascades, may not only provide molecular insights into physio-pathological activities, but also unveil novel pharmacological targets for the resolution of chronic inflammation and fibrotic disorders.

## 8. Final Remarks

Energy homeostasis is a fine-tuned process coordinating food intake, storage, and energy expenditure [1]. A disruption of this complex balance leads to fibrosis or fibrotic scarring, which can further remodel the tissue architecture through excessive buildup of extracellular matrix components [2,12] and impact on energy homeostasis [219]. Such a loop highlights the bidirectional interplay between bioenergetics and tissue injury [219]. The landscape of endogenous bioactive lipid mediators, primarily encompassing (i) EICs, (ii) SPMs, and (iii) eCBs, has emerged as a key regulatory driver that integrates inflammatory and metabolic signals to preserve tissue homeostasis. The multifaceted roles of lipid signals include the regulation of inflammatory and immune responses [220], which are crucial for preserving homeostasis, particularly in the context of energy metabolism and fibrotic progression [73,221].

Among bioactive lipids, EICs represent a broad class of compounds with opposing roles in tissue homeostasis. Indeed, their biological role is highly context-dependent, ranging from the detrimental pro-inflammatory and fibrogenic activities associated with PGF_2α_, TXA, and LTs to the anti-fibrotic and anti-inflammatory/resolving effects of PGE_2_, PGD_2_, PGI_2_, LXs, and EETs. Similarly, SPMs actively mediate the resolution of inflammation [222], are involved in the regulation of energy homeostatic processes and play a protective role in metabolic diseases and fibrotic conditions. Additionally, eCBs mediate a wide range of functions across tissues, with well-established, receptor-specific mechanisms that often lead to opposing effects in energy homeostasis and fibrosis, as demonstrated by the pro-fibrotic role of CB_1_ versus the anti-fibrotic roles of CB_2_ and TRPV1.

Against this background, an even more complex scenario is emerging where these lipid systems seem to be interconnected, thereby establishing a dynamic network of reciprocal regulation and signaling interactions. For instance, the degradative 15-PGDH enzyme catalyses the inactivation of both EICs [223] and SPMs [224]. Moreover, our group has demonstrated that the eCB AEA promotes SPM biosynthesis by enhancing the expression of their metabolic enzymes 5-LOX and 15-LOX [225]. Intriguingly, direct interaction between these lipid classes, allosteric modulation and/or relocation of their key metabolic enzymes appear to sit at the interface between their biological actions [226]. For instance, 5-LOX and 15-LOX activity may be regulated through allosteric modulation and cellular relocation induced by the phytocannabinoid cannabidiol [226]. Moreover, LXs may act as endogenous allosteric modulators of CB_1_ [227]. However, the detailed mechanisms underlying these complex crosstalks, and their functional involvement in energy homeostasis and related disorders, remain to be fully elucidated. Also to be better appreciated are the emerging interactions between dietary lipid composition, gut microbial ecology, and lipid signaling, whereby intake of specific fatty acids and gut microbiota may shape host eCB signaling by regulating lipid metabolism, inflammatory tone, and intestinal barrier integrity, while eCBs reciprocally modulate microbial composition and function [228].

## 9. Conclusions

Overall, the literature reviewed herein highlights the key role of bioactive lipid mediators in the regulation of energy homeostasis and fibrosis.

Available data point toward a fundamental role of EICs, SPMs and eCBs in the regulation of these processes under both physiological and pathological conditions, underscoring their potential as therapeutic targets. Nonetheless, clinical exploitation of compounds targeting these bioactive lipid networks still seems challenging and requires further investigation. Finally, the emerging interplay between the different bioactive compounds suggests that their effects should be considered within a more integrated network, providing a framework for the development of novel lipid-targeting therapeutic strategies.

## Figures and Tables

**Table 1:** Main roles of bioactive lipids in energy homeostasis.

Bioactive Lipid	Role in Energy Homeostasis	References	Type of Study		
			In Vitro	Animal	Human
PGE_2_	• Regulates systemic energy homeostasis and thermogenesis	[17]		√	
	• Suppresses early phase of adipocyte differentiation	[18]	√		
	• The COX-2/PGE_2_ axis exerts a dual pro- and anti-inflammatory effect in liver injury in a cell- and context-dependent way	[19,20,21]	√	√	
	• Promotes vasodilation, higher vascular permeability, and phagocytosis and facilitates immune cell recruitment	[22,23]	√	√	
	• Promotes wound healing through the antagonism of TGFβ signalling, also preventing hypertrophic scar formation		√	√	
	• Triggers ferroptosis and inhibits osteogenesis via NOS in pre-osteoblastic cells		√		
PGF_2α_	• Suppresses early phase of adipocyte differentiation	[18]	√		
	• Regulates mitochondrial dynamics and mitophagy in the bovine corpus luteum, by acting on PKC/ERK and AMPK kinases	[24]	√	√	
PGD_2_	• Activates middle-late phase of adipocyte differentiation	[18]	√		
	• Regulates cardiovascular homeostasis and protects against hypertension, atherosclerosis, and ischemic cardiac injury	[25]	√	√	√
PGI_2_	• Regulates systemic energy homeostasis and thermogenesis	[17,18]		√	
	• Activates adipogenesis	[18]	√		
	• Regulates cardiovascular homeostasis	[26]		√	√
TXA_2_	• Regulates endothelial homeostasis and angiogenesis	[26,27]	√	√	
	• Is increased in diet-induced obese mice and regulates hepatic gluconeogenesis and macrophage polarization in adipose tissue	[28]	√	√	
	• Improves insulin resistance in a calcium-dependent manner through TP receptors	[28]	√	√	
LXA_4_	• Induces browning in subcutaneous inguinal WAT and regulates thermogenesis	[29]		√	
	• Improves hepatic injury, decreases fibrosis, promotes apoptosis, and inhibits proliferation in hepatocarcinoma cells	[19]	√	√	
	• Ameliorates ischemia/reperfusion-induced acute kidney and lung injury, by inhibiting inflammation and oxidative stress and by mitigating ferroptosis, respectively	[30,31]		√	
	• Protects from LPS-induced lung injury in a rat model, by acting on neutrophils	[32]		√	
	• Exerts beneficial effects against diabetic retinopathy, by inhibiting excessive angiogenesis and pro-inflammatory mediator production	[33]		√	√
LXB_4_	• It is increased in brown adipose tissue (BAT) in young mice, suggesting a role in BAT generation	[34]		√	
LTB_4_	• Regulates insulin sensitivity and resistance	[35]	√	√	
	Promotes hepatic de novo lipogenesis via Ltb4r1 and contributes to systemic insulin resistance in obesity	[36]	√	√	
	• Promotes insulin resistance-associated inflammation via JNK pathway activation and by promoting M1 macrophage migration	[37]		√	
	• Leads to unbalanced neutrophil chemotaxis and defective wound healing in diabetic mice when produced in excessive amounts	[38]	√	√	
	• Exerts a beneficial role in influenza-induced lung inflammation by suppressing NLPR3 activation	[39]	√	√	
CysLTs	• Activate adipogenesis	[40]	√		
EETs	• Regulate brain homeostasis and cerebral blood flow	[41]		√	
20-HETE	• Regulates homeostasis and cerebral blood flow	[41]		√	
HxA	• HxA3 regulates insulin release in vitro and in vivo	[42]	√	√	
Resolvin-E series	• Regulates glucose homeostasis and insulin sensitivity	[43]		√	
	• Promotes increased production of Adiponectin in adipocytes	[5]	√	√	√
	• Regulates lipid biosynthesis in hepatocytes and exerts a hepatoprotective effect	[19]	√	√	
	• Attenuates cancer proliferation	[19,44]	√		
Resolvin-D series	• Participates in wound healing programs	[5]	√	√	√
	• Promotes increased production of adiponectin in adipocytes and reduction in leptin levels	[5]	√	√	√
	• Regulates lipid biosynthesis in hepatocytes and exerts a hepatoprotective effect	[19]	√	√	
	• Inhibits hepatocyte proliferation and is implicated in reducing cancer growth	[19,44]	√		
	• Attenuates hepatocellular carcinoma proliferation		√		
Mar1	• Regulates insulin sensitivity	[45]		√	
	• Participates in wound healing programs and tissue regeneration	[46]	√	√	
	• Regulates lipid biosynthesis in hepatocytes and exerts a hepatoprotective effect	[19]	√	√	
	• Decreases during the progression of high-fat-induced NAFLD/NASH	[47]			√
Protectins	• Promote increased production of adiponectin in adipocytes	[5]	√	√	√
n-3-DPA-derived SPMs	• Regulate adipogenesis, lipogenesis and energy expenditure	[48]		√	√
eCBs	• Regulate energy metabolism	[49,50,51,52,53,54,55,56]	√	√	√
	• Studies on CBG and PPARs agonists as therapy for altered lipid metabolism				

**Table 2:** Main roles of bioactive lipids in fibrosis.

Bioactive Lipid	Role in Fibrosis	References	Type of Study		
			In Vitro	Animal	Human
PGE_2_	• Inhibits fibroblast proliferation, myofibroblast differentiation, and inflammation	[3]	√	√	
PGF_2α_	• Facilitates pulmonary fibrosis (IPF) in a TGF-β-independent manner and by involving the adventitial fibroblasts	[57]		√	
PGD_2_	• Inhibits fibroblast proliferation, myofibroblast differentiation and inflammation	[3]	√	√	
PGI_2_	• Inhibits fibroblast proliferation, myofibroblast differentiation and inflammation	[3]	√	√	
15d-PGJ_2_	• Inhibits platelet-derived growth factor (PDGF)-induced proliferation in rat pancreatic stellate cells and NF-κB activation	[58]		√	
	• Anti-fibrotic effects in several organs via PPARγ-dependent and independent mechanisms	[59]	√	√	
TXA_2_	• Exhibits COX-1-associated pro-fibrotic effects	[3,60]		√	
	• Contributes to hepatic steatosis and inflammation via the TP receptor	[28]	√	√	
LXA_4_	• Inhibits fibroblast proliferation, myofibroblast differentiation and inflammation in multiple organs	[61,62]	√	√	
	• Ameliorates hepatic fibrosis by reducing the number of activated T cells and the release of chemokines/cytokines	[19,62]	√	√	
LXB_4_	• Exhibits anti-fibrotic effects in Shoulder rotator cuff tendon tears	[63]	√		√
LTB_4_	• Exhibits pro-fibrotic properties through TGF-β signaling and the LTB4/BLT1 axis in lungs	[64,65]	√		
CysLTs	• Exhibit pro-fibrotic effects by increasing ECM deposition	[64]		√	√
EETs	• Exhibit anti-fibrotic effects in multiple organs (e.g., kidney, heart, lung, liver)	[66]		√	
20-HETE	• Promotes renal fibrosis in mice by upregulating STAT3 pathway and increasing IL-1β, TNFα, TGFβ and collagen production	[67,68]		√	
	• Exerts pro-fibrotic effects in liver via the TGF- β1/Smad3 signal pathway, induces hepatic stellate cell activation and is increased in patients with hepatic fibrosis	[69]	√	√	√
Resolvin-E series	• Exhibits anti-fibrotic effects in multiple organs (e.g., liver, kidney)	[70,71,72,73]	√	√	
Resolvin-D series	• Exhibits anti-fibrotic effects in multiple organs (e.g., liver, kidney, heart, lung)	[72,73,74,75,76]		√	
Mar1	• Exhibits anti-fibrotic effects in multiple organs (e.g., liver, kidney, lung, heart)	[72,73,77,78,79,80,81]	√	√	
Protectins	• Exhibit anti-fibrotic effects in multiple organs	[72,73,82]		√	√
eCBs	• Exhibit pro-fibrotic effects via activation of CB_1_ in liver, lung and heart	[83,84,85,86,87,88,89]	√	√	√
	• Exert anti-fibrotic effects via activation of CB_2_ and TRPV1 in liver, lung, kidney and heart	[90,91,92,93,94,95,96,97,98,99,100,101,102,103,104,105,106,107]	√	√	√
	• Interact with CBG and PPAR agonists in the therapy of fibrosis	[108,109]	√		

## Data Availability

No new data were created or analyzed in this study. Data sharing is not applicable to this article.

## Abbreviations

The following abbreviations are used in this manuscript:

11-TXDH	11-Hydroxythromboxane B2 Reductase
12-HAEA	12-Hydroxy-N-Arachidonoylethanolamine
12-HETE-G	12-Hydroxyeicosatetraenoylglycerol
12-HpETE	12-Hydroperoxyeicosatetraenoic Acid
15-HpETE	15-Hydroperoxyeicosatetraenoic Acid
15-PGDH	15-Hydroxyprostaglandin Dehydrogenase
15d-PGJ2	15-deoxy-Δ12,14-Prostaglandin J2
17(S)-HpDHA	17(S)-Hydroperoxy-docosahexaenoic Acid
17(S)-HpDPA	17(S)-Hydroperoxy-docosapentaenoic Acid
20-HETE	20-Hydroxyeicosatetraenoic Acid
2-AG	2-Arachidonoylglycerol
5,6-EET-EA	5,6-Epoxyeicosatrienoic Acid Ethanolamide
5-HpETE	5-Hydroperoxyeicosatetraenoic Acid
5-LOX	5-Lipoxygenase
12-LOX	12-Lipoxygenase
13R-HpDPA*n*–3	13(R)-Hydroperoxy-docosapentaenoic Acid (*n*–3 DPA-derived; precursor of RvT1–4)
14-HpDPA	14-Hydroperoxy-docosapentaenoic Acid (*n*–3 DPA-derived; precursor of MaR1–3*n*–3 DPA)
14(S)-HpDHA	14(S)-Hydroperoxy-docosahexaenoic Acid
15-LOX	15-Lipoxygenase
17(S)-HDHA	17(S)-Hydroxy-docosahexaenoic Acid
18(R/S)-HpEPE	18(R/S)-Hydroperoxyeicosapentaenoic Acid
AA	Arachidonic Acid
ABHD4	Aβ-Hydrolase Domain 4 Protein
ABHD6	Aβ-Hydrolase Domain 6 Protein
ABHD12	Aβ-Hydrolase Domain 12 Protein
ACT-333679	Active Metabolite of the Prostacyclin Receptor Agonist Selexipag (see MRE-269)
AEA	N-Arachidonoylethanolamine (Anandamide)
ALX/FPR2	Formyl Peptide Receptor 2
AMPK	AMP-Activated Protein Kinase
AT-RvD1	Aspirin-Triggered Resolvin D1
BAT	Brown Adipose Tissue
BLT	Leukotriene B4 Receptor
BLT1	Leukotriene B4 Receptor 1
BLT2	Leukotriene B4 Receptor 2
BMI	Body Mass Index
CB1	Cannabinoid Receptor 1
CB2	Cannabinoid Receptor 2
CBG	Cannabigerol
CF	Cystic Fibrosis
ChemR23	Chemerin Receptor 23
COVID-19	Coronavirus Disease 2019
COX	Cyclooxygenase
COX-1	Cyclooxygenase-1
COX-2	Cyclooxygenase-2
CYP450	Cytochrome P450
CysLTs	Cysteinyl Leukotrienes
CysLTR1	Cysteinyl Leukotriene Receptor 1
CysLTR2	Cysteinyl Leukotriene Receptor 2
DAG	Diacylglycerol
DAGL	Diacylglycerol Lipase
DAGL-α/β	Diacylglycerol Lipase Alpha/Beta
DHA	Docosahexaenoic Acid
DHEA	N-Docosahexaenoylethanolamine (Synaptamide)
DP	Prostaglandin D Receptor
DPA	Docosapentaenoic Acid
eCBs	Endocannabinoids
ECM	Extracellular Matrix
ECS	Endocannabinoid System
EETs	Epoxyeicosatrienoic Acids
EP	Prostaglandin E Receptor
EPA	Eicosapentaenoic Acid
EPEA	N-Epoxyeicosatetraenoylethanolamine
EPHXs	Epoxide Hydrolases
ERK	Extracellular Signal-Regulated Kinase
EtNH2	Ethanolamine
FAAH	Fatty Acid Amide Hydrolase
FP	Prostaglandin F Receptor
GPCR	G Protein-Coupled Receptor
GPR18	G Protein-Coupled Receptor 18
GPR32	G Protein-Coupled Receptor 32
GPR37	G Protein-Coupled Receptor 37
GPR55	G Protein-Coupled Receptor 55
GPR119	G Protein-Coupled Receptor 119
GPx4	Glutathione Peroxidase 4
HETE	Hydroxyeicosatetraenoic Acid
HpDHA	Hydroperoxy-Docosahexaenoic Acid
HpDPA	Hydroperoxy-Docosapentaenoic Acid
HpEPE	Hydroperoxyeicosapentaenoic Acid
HpETE	Hydroperoxyeicosatetraenoic Acid
HSCs	Hepatic Stellate Cells
HxA	Hepoxilin A
HxA3	Hepoxilin A3
HxB	Hepoxilin B
IBD	Inflammatory Bowel Disease
IL-1β	Interleukin-1 Beta
IP	Prostacyclin I Receptor
IPF	Idiopathic Pulmonary Fibrosis
JNK	c-Jun N-terminal Kinase
LEA	N-Linoleoylethanolamine
LGR6	Leucine-Rich Repeat-Containing G-Protein Coupled Receptor 6
LOX	Lipoxygenase
LPS	Lipopolysaccharide
LT	Leukotriene
LTA4	Leukotriene A4
LTB4	Leukotriene B4
LTB5	Leukotriene B5
LTC4	Cysteinyl Leukotriene C4
LTD4	Cysteinyl Leukotriene D4
LTE4	Cysteinyl Leukotriene E4
LX	Lipoxin
LXA4	Lipoxin A4
LXB4	Lipoxin B4
Lyso-NArPE	Lyso-N-Arachidonoyl Phosphatidylethanolamine
Lyso-PLD	Lysophospholipase D
MAGL	Monoacylglycerol Lipase
MaR1	Maresin 1
MaR2	Maresin 2
MaR*n*–3 DPA	Maresins, *n*–3 DPA-derived
MaR1-3*n*–3 DPA	Maresins 1–3, *n*–3 DPA-derived
MCTR1-3	Maresin Conjugates in Tissue Regeneration 1–3
MRE-269	Active Metabolite of the Prostacyclin Receptor Agonist Selexipag
MRI-1867	Peripherally Restricted Cannabinoid Receptor 1 Antagonist
*n*–3 DPA	*N*–3 Docosapentaenoic Acid
NAAA	*N*-Acylethanolamine Acid Amidase
NAFLD	Non-Alcoholic Fatty Liver Disease
NAPE-PLD	N-Acylphosphatidylethanolamines-Hydrolyzing Phospholipase D
NArPE	N-Arachidonoyl Phosphatidylethanolamine
NASH	Non-Alcoholic Steatohepatitis
NF-κB	Nuclear Factor Kappa-Light-Chain-Enhancer of Activated B Cells
NLRP3	NOD-, LRR- and Pyrin Domain-Containing Protein 3
NPD1	Neuroprotectin D1
NSAIDs	Non-Steroidal Anti-Inflammatory Drugs
OEA	N-Oleoylethanolamine
ONO-1301	Synthetic Prostacyclin (PGI2) Analogue
oxoETEs (12-oxoETEs)	Oxo-Eicosatetraenoic Acids
p-AEA	Phospho-N-Arachidonoylethanolamine
PCTRs	Protectin Conjugates in Tissue Regeneration
PD1	Protectin D1
PDGF	Platelet-Derived Growth Factor
PDX	Protectin DX
PD*n*–3 DPA	Protectins, *n*–3 DPA-derived
PD1–2*n*–3 DPA	Protectins D1–2, *n*–3 DPA-derived
PEA	N-Palmitoylethanolamine
PG	Prostaglandin
PGD2	Prostaglandin D2
PGDS	Prostaglandin D2 Synthase
PGE2	Prostaglandin E2
PGE2-G	Prostaglandin E2 Glycerol
PGE3	Prostaglandin E3
PGES	Prostaglandin E Synthase
PGF2α	Prostaglandin F2α
PGFS	Prostaglandin F Synthase
PGH2	Prostaglandin H2
PGI	Prostacyclin
PGI2	Prostacyclin I2
PGI3	Prostacyclin I3
PGIS	Prostacyclin I Synthase
PGJ2	Prostaglandin J2
PI3K/Akt	Phosphoinositide 3-Kinase/Protein Kinase B
PKC	Protein Kinase C
PL	Phospholipase
PLA2	Phospholipase A2
PLC	Phospholipase C
POEA	N-Palmitoleoylethanolamine
PPARα/γ/δ	Peroxisome Proliferator-Activated Receptor α/γ/δ
PTGR	Prostaglandin Reductase
PUFA	Polyunsaturated Fatty Acid
RIF	Radiation-Induced Fibrosis
RvD	D-Series Resolvins
RvD1	Resolvin D1
RvD2	Resolvin D2
RvD3	Resolvin D3
RvD4	Resolvin D4
RvD5	Resolvin D5
RvD6	Resolvin D6
RvD*n*–3 DPA	D-Series Resolvins, *n*–3 DPA-derived
RvD1–2*n*–3 DPA	Resolvins D1–2, *n*–3 DPA-derived
RvD5*n*–3 DPA	Resolvin D5, *n*–3 DPA-derived
RvE	E-Series Resolvins
RvE1	Resolvin E1
RvE2	Resolvin E2
RvE3	Resolvin E3
RvT1-4	13-Series Resolvins 1–4
RvTs	13-Series Resolvins
SEA	N-Stearoylethanolamine
sEH	Soluble Epoxide Hydrolase
SIRT1	Sirtuin 1
Smad(s)	Small Mothers Against Decapentaplegic
SPMs	Specialized Pro-Resolving Mediators
STAT3	Signal Transducer and Activator of Transcription 3
TG	Triglyceride
TGF	Transforming Growth Factor
THC	Δ9-Tetrahydrocannabinol
TNFα	Tumor Necrosis Factor Alpha
TP	Thromboxane Receptor
TRPV1	Transient Receptor Potential Vanilloid Type-1
TXA2	Thromboxane A2
TXA3	Thromboxane A3
TXAS	Thromboxane Synthase
TXs	Thromboxanes
WAT	White Adipose Tissue
α-SMA	Alpha-Smooth Muscle Actin
